# Physical activity and all-cause mortality across levels of overall and abdominal adiposity in European men and women: the European Prospective Investigation into Cancer and Nutrition Study (EPIC)^1^,^2^,^3^,^4^,^5^,^6^

**DOI:** 10.3945/ajcn.114.100065

**Published:** 2015-01-14

**Authors:** Ulf Ekelund, Heather A Ward, Teresa Norat, Jian’an Luan, Anne M May, Elisabete Weiderpass, Stephen J Sharp, Kim Overvad, Jane Nautrup Østergaard, Anne Tjønneland, Nina Føns Johnsen, Sylvie Mesrine, Agnès Fournier, Guy Fagherazzi, Antonia Trichopoulou, Pagona Lagiou, Dimitrios Trichopoulos, Kuanrong Li, Rudolf Kaaks, Pietro Ferrari, Idlir Licaj, Mazda Jenab, Manuela Bergmann, Heiner Boeing, Domenico Palli, Sabina Sieri, Salvatore Panico, Rosario Tumino, Paolo Vineis, Petra H Peeters, Evelyn Monnikhof, H Bas Bueno-de-Mesquita, J Ramón Quirós, Antonio Agudo, María-José Sánchez, José María Huerta, Eva Ardanaz, Larraitz Arriola, Bo Hedblad, Elisabet Wirfält, Malin Sund, Mattias Johansson, Timothy J Key, Ruth C Travis, Kay-Tee Khaw, Søren Brage, Nicholas J Wareham, Elio Riboli

**Affiliations:** 1 From the Medical Research Council (MRC) Epidemiology Unit, University of Cambridge, United Kingdom (UE, JL, SJS, SB, and NJW); the Department of Sport Medicine, Norwegian School of Sport Sciences, Oslo, Norway (UE); Imperial College, London, United Kingdom (HAW, TN, PV, HBB-d-M, and ER; University Medical Centre Utrecht, Julius Centre for Health Sciences and Primary Care, Utrecht, The Netherlands (AMM, PHP, and EM); the Department of Community Medicine, Faculty of Health Sciences, University of Tromsø, Tromsø, Norway (EW); the Department of Research, Cancer Registry of Norway, Oslo, Norway (EW); the Department of Medical Epidemiology and Biostatistics, Karolinska Institutet, Stockholm, Sweden (EW); Samfundet Folkhälsan, Helsinki, Finland (EW); the Section for Epidemiology, Department of Public Health, Aarhus University, Aarhus, Denmark (KO and JNØ); the Department of Cardiology, Center for Cardiovascular Research, Aalborg University Hospital, Aalborg, Denmark (KO and JNØ); Danish Cancer Society, Copenhagen, Denmark (A Tjønneland and NFJ); Inserm, Centre for Research in Epidemiology and Population Health, Nutrition, Hormones and Women’s Health team, Villejuif, France (SM, AF, and GF); the Univeristy of Paris Sud, UMRS 1018, Villejuif, France (SM, AF, and GF); IGR, Villejuif, France (SM, AF, and GF); WHO Collaborating Center for Food and Nutrition Policies, Department of Hygiene, Epidemiology and Medical Statistics, University of Athens Medical School, Athens, Greece (A Trichopoulou and PL); Hellenic Health Foundation, Athens Greece (A Trichopoulou and DT); the Department of Epidemiology, Harvard School of Public Health, Boston, MA (PL and DT); the Bureau of Epidemiologic Research, Academy of Athens, Athens, Greece (PL and DT); the Division of Cancer Epidemiology, German Cancer Research Centre, Heidelberg, Germany (KL and RK); International Agency for Research on Cancer (IARC), Lyon, France (PF, IL, and MJ); the Department of Epidemiology, Deutsches Institut für Ernährungsforschung, Potsdam-Rehbrücke, Germany (MB and HB); Molecular and Nutrional Epidemiology Unit, ISPO, Cancer Prevention and Research Institute, Florence, Italy (DP); Epidemiology and Prevention Unit, Fondazione IRCCS Istituto Nazionale dei Tumori, Milano, Italy (SS); Dipartimento di Medicina Clinica e Chirurgia, Federico ii University, Naples, Italy (SP); UOS Registro Tumori e UOC Anatomia Patologica, Ospedale “Civile MP Arezzo” ASP 7, Ragusa, Italy (RT); HuGEF Foundation, Turin, Italy (PV); National Institute for Public Health and the Environment, Bilthoven, The Netherlands (HBB-d-M); the Department of Gastroenterology and Hepatology, University Medical Centre, Utrecht, The Netherlands (HBB-d-M); Public Health Directorate, Asturias, Spain (JRQ); Unit of Nutrition, Environment and Cancer, Cancer Epidemiology Research Program, Catalan Institute of Oncology, Barcelona, Spain (AA); Andalusian School of Public Health, Granada, Spain (M-JS); CIBER de Epidemiología y Salud Pública (CIBERESP), Spain (M-JS, JMH, and EA); the Department of Epidemiology, Murcia Regional Health Council, Murcia, Spain (JMH); Navarre Public Health Institute, Pamplona, Spain (EA); Public Health Division of Gipuzkoa, Instituto BIO-Donostia, Basque Government, CIBER Epidemiología y Salud Pública-CIBERESP, Spain (LA); Cardiovascular Epidemiology (BH) and Nutritional Epidemiology (EW), Department of Clinical Sciences, Lund University, Malmö, Sweden; the Department of Public Health and Clinical Medicine, Umeå University, Umeå, Sweden (MS and MJ); Cancer Epidemiology Unit, Nuffield Department of Clinical Medicine, University of Oxford, Oxford, United Kingdom (TJK and RCT); and Clinical Gerontology Unit, University of Cambridge, Cambridge, United Kingdom (K-TK).

**Keywords:** cohort study, epidemiology, obesity, physical activity, exercise, mortality, population attributable fraction

## Abstract

**Background:** The higher risk of death resulting from excess adiposity may be attenuated by physical activity (PA). However, the theoretical number of deaths reduced by eliminating physical inactivity compared with overall and abdominal obesity remains unclear.

**Objective:** We examined whether overall and abdominal adiposity modified the association between PA and all-cause mortality and estimated the population attributable fraction (PAF) and the years of life gained for these exposures.

**Design:** This was a cohort study in 334,161 European men and women. The mean follow-up time was 12.4 y, corresponding to 4,154,915 person-years. Height, weight, and waist circumference (WC) were measured in the clinic. PA was assessed with a validated self-report instrument. The combined associations between PA, BMI, and WC with mortality were examined with Cox proportional hazards models, stratified by center and age group, and adjusted for sex, education, smoking, and alcohol intake. Center-specific PAF associated with inactivity, body mass index (BMI; in kg/m^2^) (>30), and WC (≥102 cm for men, ≥88 cm for women) were calculated and combined in random-effects meta-analysis. Life-tables analyses were used to estimate gains in life expectancy for the exposures.

**Results:** Significant interactions (PA × BMI and PA × WC) were observed, so HRs were estimated within BMI and WC strata. The hazards of all-cause mortality were reduced by 16–30% in moderately inactive individuals compared with those categorized as inactive in different strata of BMI and WC. Avoiding all inactivity would theoretically reduce all-cause mortality by 7.35% (95% CI: 5.88%, 8.83%). Corresponding estimates for avoiding obesity (BMI >30) were 3.66% (95% CI: 2.30%, 5.01%). The estimates for avoiding high WC were similar to those for physical inactivity.

**Conclusion:** The greatest reductions in mortality risk were observed between the 2 lowest activity groups across levels of general and abdominal adiposity, which suggests that efforts to encourage even small increases in activity in inactive individuals may be beneficial to public health.

## INTRODUCTION

Physical inactivity has been consistently associated with an increased risk of all-cause mortality independent of general adiposity defined by BMI (1–3). Studies that have examined the combined associations between physical activity (PA),^7^ BMI, and mortality suggest that PA protects again premature death but does not eliminate the increased risk associated with high BMI (4–8). However, these previous examinations of the combined association between PA and obesity with mortality have relied on self-reported anthropometric data (5–7), have been restricted to single sex cohorts (6, 8), and have included only small numbers of deaths (8, 9), and few studies have examined PA combined with both BMI and waist circumference (WC) in relation to mortality (5, 8, 9). Furthermore, those that have examined the combined associations between PA, adiposity, and mortality have used a dichotomous categorization of PA and BMI (9), leaving uncertainty about whether PA protects against premature deaths across established BMI and WC categories (10, 11).

Whereas it could be hypothesized that PA exerts its influence on mortality indirectly through reducing adiposity, recent data from the European Prospective Investigation into Cancer and Nutrition (EPIC) suggest that PA is unrelated to change in body weight and inversely, albeit weakly, associated with change in WC (12). Thus, PA may interact differentially with BMI and WC in relation to all-cause mortality.

We therefore examined the associations between PA and all-cause mortality and whether BMI and WC modified these associations in a large sample of 334,161 men and women followed for >12 y from the EPIC study, in which both BMI and WC were measured during clinical examinations at baseline. As a secondary aim we estimated how many deaths could theoretically be avoided if inactive or obese individuals were more active or nonobese, respectively, and calculated the years of gain in life expectancy from avoiding physical inactivity, high BMI (in kg/m^2^; ≥30), and high WC (>88 cm and >102 cm in women and men), separately and combined in the cohort.

## METHODS

### The EPIC cohort

EPIC is a multicenter prospective cohort study, which recruited 519,978 volunteers from 23 centers in 10 countries [Sweden, Denmark, Norway, The Netherlands, United Kingdom, France, Germany, Spain, Italy, and Greece] between 1992 and 2000. The study population included volunteers aged mostly 25–70 y at the time of recruitment and has been described in detail previously (13, 14). There were 518,408 participants for whom vital statistics were available at the end of follow-up (2010). Individuals who reported either baseline heart disease (*n* = 6256), stroke (*n* = 3485), cancer (*n* = 15,926) or a combination of these conditions (*n* = 1092) were excluded from the analysis. Participants who were missing data on PA were excluded (*n* = 45,725); this included the entire cohort from Norway (*n* = 36,920) as the information collected on leisure-time PA was not compatible with the other EPIC centers questionnaires. Participants in the top and bottom 0.5th percentile of the energy intake to estimated basal metabolic rate ratio were excluded (*n* = 8637) because of unrealistic dietary intake information, as were those with missing data on the following covariates: height and weight (*n* = 54,522), WC (*n* = 28,548), alcohol (*n* = 478), education (*n* = 12,276), and smoking (*n* = 3031). Participants with extreme anthropometric measurements (height <130 cm, weight >250 kg, BMI <18.5, WC <40 cm, WC >160 cm, or BMI >25 and WC <60 cm) were additionally excluded (*n* = 4271). The current analysis therefore included all participants (*n* = 334,161) for whom measured height, weight, and WC were available. A detailed comparison between excluded and included participants is available elsewhere (**Supplemental Table 1**).

To maintain sample size per center without combining groups considered to be too heterogeneous with respect to lifestyle characteristics, the 23 EPIC centers were analyzed as the following 11 groups in the current analysis: France, Italy, Spain, United Kingdom health conscious (Oxford participants recruited through the Vegetarian Society), United Kingdom general (all United Kingdom participants not included in the Health Conscious group), The Netherlands, Greece, Heidelberg, Potsdam, Sweden, and Denmark (14). The study was approved by the Institutional Review Board at the International Agency for Research on Cancer and local ethics committees, and informed consent forms were signed at each local center.

### Assessment of physical activity

Data on occupational, recreational, and household PA during the past year either were obtained from an in-person interviews or were self-administered by using a standardized questionnaire. Participants reported their level of occupational PA as either sedentary (e.g., office work), standing (e.g., hairdresser, guard), physical work (e.g., plumber, nurse), or heavy manual work (e.g., construction worker, bricklayer). The Cambridge Index of PA was derived by combining occupational activity level with recreational activity, as assessed by the amount of time in hours per week during winter and summer spent cycling and in other physical exercises (e.g., jogging, swimming) and is summarized into 4 groups: active, moderately active, moderately inactive, and inactive (15, 16).

We examined the validity of the Cambridge Index in an independent substudy in 1941 men and women similar in age to those in the original EPIC cohort, across all 10 EPIC countries by using combined movement and heart rate monitoring as the criterion (16). PA energy expenditure (PAEE) increased significantly by increasing categories of self-reported PA (*P-*trend < 0.0001), with a significant correlation between measured PAEE and the categorical PA index (Spearman correlation = 0.33, *P* < 0.013). Further calibration results suggest that the average PAEE across categories of PA were as follows: inactive (36 kJ/kg daily); moderately inactive (41 kJ/kg daily); moderately active (46 kJ/kg daily), and active (51 kJ/kg daily). The format of the PA questions was somewhat different in Naples (Italy) relative to the other centers in the current analysis; however, the data from Naples were transformed for inclusion in the PA index. In Umeå (Sweden), a 4-level PA variable was derived from cross-tabulation of occupational and exercise; owing to the similarity with the Cambridge categories (inactive, moderately inactive, moderately active, active) (16), the Umeå classification has been incorporated into the current analysis.

### Assessment of anthropometric measures

Body weight (in kg) and height (in cm) were measured at baseline according to standardized procedures without shoes (17). Self-reported anthropometric data (Oxford) were adjusted by using prediction equations derived from the general population, where a subset of participants had both self-reported and measured anthropometric data available (17, 18). WC (in cm) was measured at the narrowest torso circumference or at the midpoint between the lower ribs and iliac crest. Weight measurements were corrected to account for protocol differences between centers as previously described (18). BMI was calculated as body weight (in kg) divided by height squared (in m). Individuals were categorized into normal-weight (BMI: 18.5–24.9), overweight (BMI: 25–30), and obese (BMI ≥30). Participants were dichotomized by WC values by using the cutoffs of ≥102 cm among men and ≥88 cm among women.

### Assessment of endpoints

Mortality data were obtained at the regional or national level. In Denmark, Italy, The Netherlands, Spain, Sweden, and the United Kingdom, vital status and the causes and the dates of death were ascertained by death indexes, cancer registry records, and national health statistics. Active follow-up was adopted in Germany, Greece, and France. Causes of death were coded according to the International Classification of Diseases, 10th Revision (19). The endpoint in the current analysis was death from all causes collected between 2008 and 2010, depending on the center.

### Statistical analysis

We examined the associations between PA, adiposity, and all-cause mortality using Cox regression models to obtain HRs. The baseline hazard function of the models was stratified by center and age, with age categorized as <25 y, then every 5-y age group, and ≥75 y. Significant interactions (PA × BMI and PA × WC) were observed with respect to all-cause mortality (*P* < 0.005). HRs were therefore estimated within strata defined by BMI (3 groups according to WHO classification) and WC (2 groups, cutoffs: ≥102 cm for men and ≥88 cm for women). Two sets of covariates were included in the models: *1*) sex; *2*) sex, lifestyle (alcohol intake and smoking), and demographic covariates (education). The lifestyle and demographic characteristics selected a priori were education (none/primary school, technical/professional, secondary, and longer education), alcohol intake (baseline: 0, >0–6, >6–12, >12–24, >24–60, and >60 g/d), and smoking (current, former, and never). In addition to the main covariates described above, potential dietary confounders were evaluated for inclusion in the Cox regression models. However, none of the dietary variables tested [fiber (g/d), energy (kcal/d), dairy (g/d), red meat (g/d), and fish (g/d); crude and adjusted for energy intake by using both the standard and residual methods] yielded an important change (<10%) in the HR estimates for the PA and adiposity exposure variables and were therefore not included. Similarly, stratification of the highest BMI group into 2 groups (30–34.9 and ≥35) yielded similar estimates in each group and are therefore not presented.

We estimated center-specific RRs by comparing levels of physical inactivity, general and abdominal obesity—adjusted for sex, education, smoking, and alcohol intake—using binomial regression. We also estimated RRs adjusted for BMI. Adjusted RRs were used to calculate the population attributable fraction (PAF):


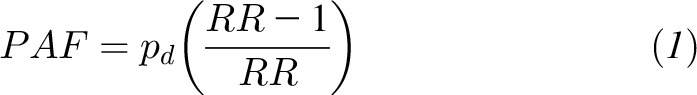


Where *p_d_* is the proportion of deaths exposed to the risk factor of interest, and *RR* is the adjusted RR (20). The STATA command “punafcc” was used to calculate the PAFs and 95% CIs. PAFs were thereafter combined by using a random-effect meta-analysis to assess the proportion of mortality that could have been averted by avoiding the following risk factors: inactivity (all inactive individuals become at least moderately inactive, i.e., moving from category 1 to category 2 or higher of the Cambridge Index), high BMI [all individuals classified as obese (BMI ≥30) become nonobese], and high WC (≥88 cm and ≥102 cm in women and men, respectively). These analyses were adjusted for the same covariates used above. Gain in life expectancy was calculated from life tables (21).

Analyses within subgroups defined by sex, age group, and smoking status and a sensitivity analysis that excluded the first 3 y of follow-up (3116 participants were excluded, of whom 2317 were deceased)—to minimize the possibility of reverse causation due to underlying disease—were performed. All analyses were performed by using STATA 12 statistical software (StataCorp LP).

## RESULTS

A total of 116,980 men (mean age 52.6 y) and 217,181 women (mean age 51.2 y) were included in the current analysis (**Tables 1** and **2**; Supplemental Table 1 and** Supplemental Table 2**). Across all centers, the mean follow-up time was 12.4 y, corresponding to 4,154,915 person-years. There were 11,086 deaths among men and 10,352 deaths among women.

**TABLE 1:** Sample size, length of follow-up, age at recruitment, and frequency of total and cause-specific mortality in the EPIC, by country and study center^1^

	Total participants, *n*		Age at recruitment,^2^ y		Mean person-years follow-up		Mortality rate per 1000 person-years^3^		Inactive, %		Moderately inactive, %		Moderately active, %		Active, %	
EPIC center	M	F	M	F	M	F	M	F	M	F	M	F	M	F	M	F
France		17,099		52.8 ± 6.5		15.1		3.6		17.1		41.0		32.9		9.0
Italy	12,533	29,697	50.3 ± 7.5	50.7 ± 8.1	12.6	12.1	6.8	4.7	13.5	36.9	36.0	39.1	23.8	14.9	26.7	9.2
Spain	14,763	24,355	50.7 ± 7.2	48.3 ± 8.3	13.5	13.7	6.2	5.0	21.3	48.5	30.0	35.2	27.2	12.1	21.6	4.2
United Kingdom																
General	9931	13,164	58.4 ± 9.3	57.0 ± 9.3	13.1	13.6	11.1	7.6	33.8	34.2	27.9	35.5	20.6	19.3	17.7	11.1
Health conscious	7989	25,562	43.2 ± 12.9	41.0 ± 12.3	12.6	12.6	7.7	5.3	16.5	15.8	33.8	38.1	25.0	27.4	24.7	18.8
Netherlands	7140	23,142	43.0 ± 11.0	51.8 ± 11.2	12.6	12.9	8.2	7.0	8.5	7.5	22.7	26.3	24.7	26.9	44.2	39.3
Greece	9747	14,527	52.4 ± 12.7	53.6 ± 12.3	9.3	9.9	10.9	4.6	33.0	54.0	26.5	25.9	26.7	16.1	13.9	4.0
Germany																
Heidelberg	10,608	12,144	52.2 ± 7.1	49.2 ± 8.6	11.3	11.4	10.3	5.8	10.3	11.9	33.8	35.9	29.1	28.8	27.0	23.3
Potsdam	9836	15,099	52.1 ± 8.0	49.0 ± 9.3	11.2	11.3	10.0	6.0	21.5	21.6	36.2	39.3	24.6	23.9	17.7	15.2
Sweden	9480	14,544	58.7 ± 7.0	57.1 ± 7.8	13.9	14.1	13.9	8.3	21.5	22.3	38.1	38.4	22.9	23.8	17.4	15.5
Denmark	24,953	27,848	56.5 ± 4.3	56.7 ± 4.4	11.5	11.8	11.7	7.3	11.1	10.3	28.8	32.1	23.9	25.1	36.3	32.5
Total	116,980	217,181	52.6 ± 9.6	51.2 ± 10.3	11.1	12.6	11.5	6.3	18.2	25.2	31.2	35.1	24.8	22.4	25.7	17.4

1 EPIC, European Prospective Investigation into Cancer and Nutrition.

2 Values are means ± SDs.

3 Five-year age-standardized death rates (in the European standard population) were computed for the common age range of 50–69 y.

**TABLE 2:** Anthropometric, lifestyle, and demographic characteristics of the EPIC cohort across levels of physical activity, by sex^1^

	Men					Women				
	Total *N*	Inactive, %	Moderately inactive, %	Moderately active, %	Active, %	Total *N*	Inactive, %	Moderately inactive, %	Moderately active, %	Active, %
BMI										
18.5–24.9 kg/m^2^	40,006	28.7	34.4	35.1	37.1	113,216	37.5	54.1	59.5	59.9
25–29.9 kg/m^2^	58,005	50.2	49.9	49.6	49.1	69,981	36.8	32.0	29.2	29.8
30–34.9 kg/m^2^	16,290	17.7	13.6	13.4	12.1	25,196	18.2	10.5	8.7	8.0
>35 kg/m^2^	2,629	3.4	2.2	2.0	1.8	8,788	7.5	3.4	2.6	2.2
Waist circumference (cm)										
<88 (F)/<102 (M)	89,938	68.0	76.6	78.7	81.7	164,928	63.3	78.1	81.9	82.1
≥88 (F)/≥102 (M)	27,042	32.0	23.4	21.3	18.3	52,253	36.8	21.9	18.1	17.8
Alcohol										
0 g/d	7646	10.9	5.7	5.6	5.3	37,317	30.5	15.2	11.4	9.4
>0–6 g/d	23,823	24.0	20.0	18.9	19.7	87,963	38.2	41.0	41.4	41.6
>6–12 g/d	18,952	15.6	16.5	16.2	16.2	39,013	13.3	18.3	20.1	21.3
>12–24 g/d	26,002	19.5	23.2	22.9	22.3	32,427	11.1	15.6	16.8	16.8
>24–60 g/d	31,802	23.2	27.7	28.7	28.0	19,064	6.4	9.3	9.7	10.1
>60 g/d	8755	6.9	6.9	7.7	8.5	1,397	0.5	0.7	0.7	0.8
Smoking										
Never	36,836	28.1	32.1	32.3	32.3	124,251	63.4	57.1	55.6	50.7
Former	43,575	38.0	37.6	36.9	36.7	48,851	16.5	22.8	25.1	27.4
Smoker	36,569	33.9	30.3	30.7	31.1	44,079	20.2	20.2	19.4	21.9
Education										
None/primary school	41,129	39.9	29.4	35.6	38.4	76,142	54.7	31.9	25.6	25.2
Technical/professional	28,908	21.4	22.6	24.8	29.6	54,216	17.2	25.3	26.6	33.5
Secondary	13,702	12.0	13.6	11.0	9.9	38,295	13.9	18.6	19.8	18.4
Longer education	33,241	26.8	34.4	28.7	22.1	48,528	14.2	24.2	28.0	23.0

1 EPIC, European Prospective Investigation into Cancer and Nutrition.

Within the BMI strata, the hazard of all-cause mortality was reduced by 20–30% across groups when the moderately inactive individuals were compared with the inactive individuals (the reference group). In normal-weight and overweight individuals, higher levels of PA were associated with further reduction in hazards, which were most pronounced in the normal-weight group, i.e., decreased by 41% in those categorized as active compared with those categorized as inactive. In contrast, in those with a BMI >30, no further reduction in hazard was observed with increasing levels of PA beyond that for the moderately inactive group. Adjustment for additional covariates did not materially change these estimates (model 2; **Table 3**).

**TABLE 3:** HRs and 95% CIs of all-cause mortality in relation to physical activity levels within strata of BMI and waist circumference groups^1^

	Deaths, *n*	Inactive	Moderately inactive	Moderately active	Active	HR per one-level difference in physical activity^2^
BMI						
Model 1^3^						
18.5–24.9 kg/m^2^	8285	1 (reference)	0.70 (0.66, 0.74)	0.64 (0.60, 0.69)	0.59 (0.55, 0.63)	0.84 (0.82, 0.86)
25–29.9 kg/m^2^	8815	1 (reference)	0.77 (0.74, 0.82)	0.74 (0.70, 0.79)	0.72 (0.67, 0.77)	0.90 (0.88, 0.92)
>30 kg/m^2^	4338	1 (reference)	0.80 (0.74, 0.87)	0.73 (0.67, 0.81)	0.79 (0.71, 0.87)	0.91 (0.88, 0.94)
Model 2^4^						
18.5–24.9 kg/m^2^	8285	1 (reference)	0.76 (0.72, 0.81)	0.71 (0.67, 0.76)	0.65 (0.60, 0.70)	0.87 (0.85, 0.89)
25–29.9 kg/m^2^	8815	1 (reference)	0.82 (0.77, 0.86)	0.78 (0.73, 0.83)	0.75 (0.70, 0.80)	0.91 (0.89, 0.93)
>30 kg/m^2^	4338	1 (reference)	0.84 (0.78, 0.91)	0.76 (0.69, 0.84)	0.82 (0.74, 0.90)	0.92 (0.89, 0.95)
Waist circumference (cm)						
** **Model 1^3^						
<88 (F)/<102 (M)	14,362	1 (reference)	0.75 (0.72, 0.78)	0.70 (0.67, 0.74)	0.67 (0.63, 0.70)	0.88 (0.86, 0.89)
≥88 (F)/≥102 (M)	7076	1 (reference)	0.79 (0.75, 0.84)	0.74 (0.69, 0.80)	0.76 (0.70, 0.82)	0.90 (0.88, 0.92)
Model 2^4^						
<88 (F)/<102 (M)	14,362	1 (reference)	0.80 (0.76, 0.83)	0.76 (0.72, 0.79)	0.71 (0.68, 0.75)	0.90 (0.88, 0.91)
≥88 (F)/≥102 (M)	7076	1 (reference)	0.84 (0.79, 0.89)	0.78 (0.73, 0.84)	0.80 (0.73, 0.86)	0.91 (0.89, 0.94)

1 Data were analyzed by Cox regression models.

2 Physical activity variables entered into the model as an ordinal variable.

3 Model 1: adjusted for sex; stratified by age at recruitment and study center. For waist circumference, sex was included as a stratum variable rather than as a covariate to meet the proportional hazards assumption.

4 Model 2: adjusted as for model 1 and for education, smoking, and alcohol.

Similar results were observed when participants were stratified according to abdominal adiposity (WC ≥88 cm and ≥102 cm in women and men, respectively). The most pronounced decreased hazard was observed between the inactive (reference) and moderately inactive groups in both the abdominally lean (HR: 0.75; 95% CI: 0.72, 0.78) and abdominally obese (HR: 0.79; 95% CI: 0.75, 0.84) groups. A further reduction in hazard across PA groups was observed in abdominally lean but not in abdominally obese groups (Table 3). Results were similar within subgroups defined by sex, age, and smoking status (**Supplemental Tables 3–5**).

Similar to overall activity, higher levels of recreational activity was associated with lower HRs of all-cause mortality independent of covariates in each BMI and WC group; however, occupational activity was not related to mortality in working individuals (**Supplemental Table 6**).

**FIGURE 1:**
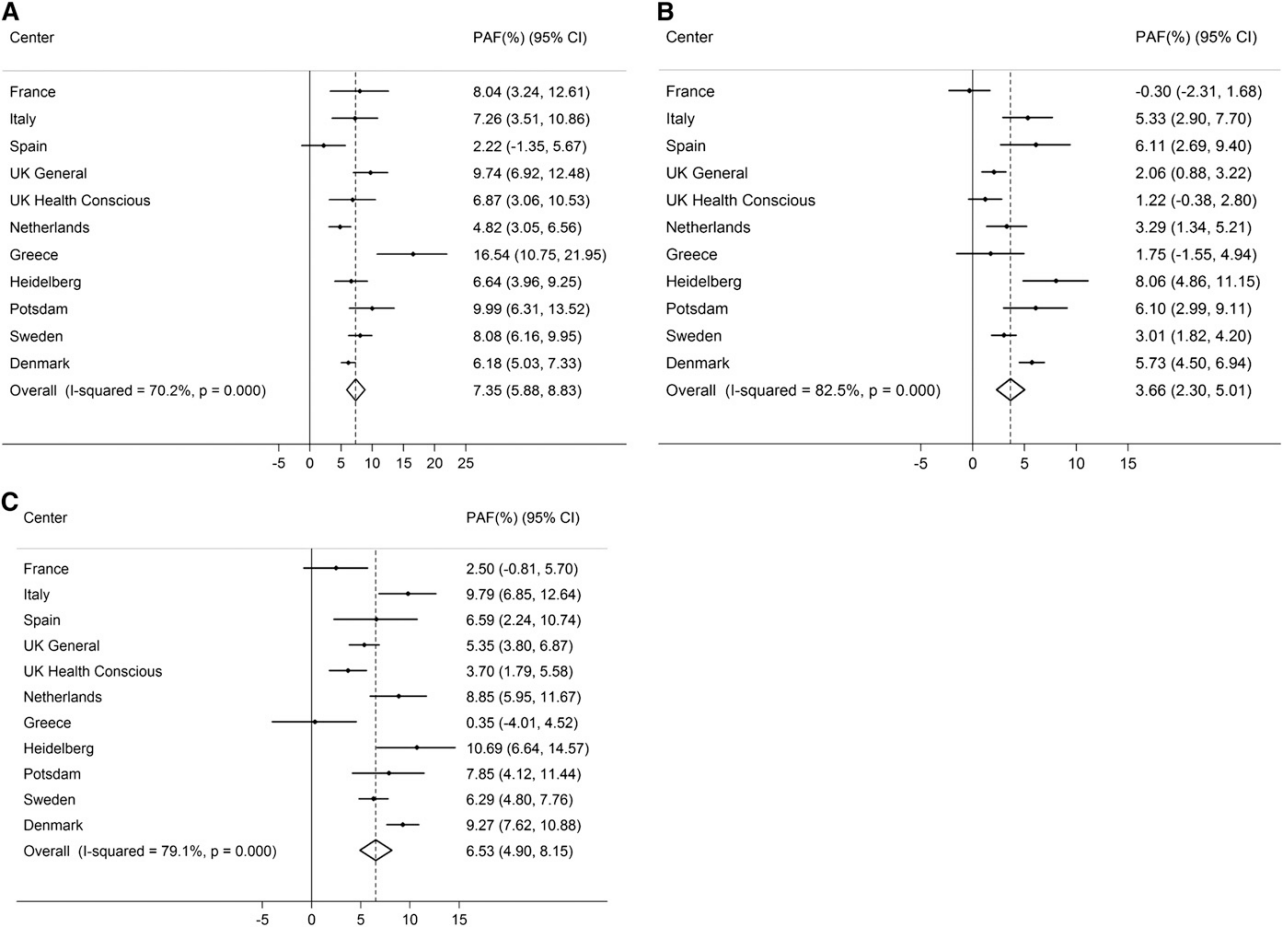
Proportion of deaths averted when all inactivity (lowest category of the Cambridge Index; A), general obesity [BMI (in kg/m^2^) >30; B], and abdominal obesity (≥88 cm and ≥102 cm in men and women, respectively; C) were removed. Data were adjusted for age, sex, education, smoking, and alcohol intake (*n* = 334,161). PAF, population attributable fraction.

**FIGURE 2:**
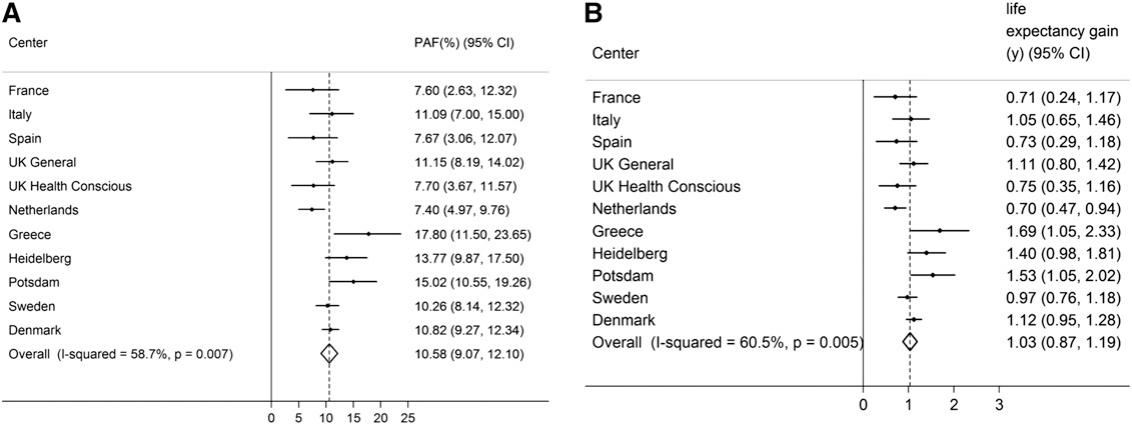
The combined proportion of number of deaths theoretically averted when all inactivity (lowest category of Cambridge Index) and general obesity [BMI (in kg/m^2^) >30] were removed (A) and estimated life expectancy gain when all inactivity and general obesity were avoided (B). Data were adjusted for age, sex, education, smoking, and alcohol intake (*n* = 334,161). PAF, population attributable fraction.

**FIGURE 3:**
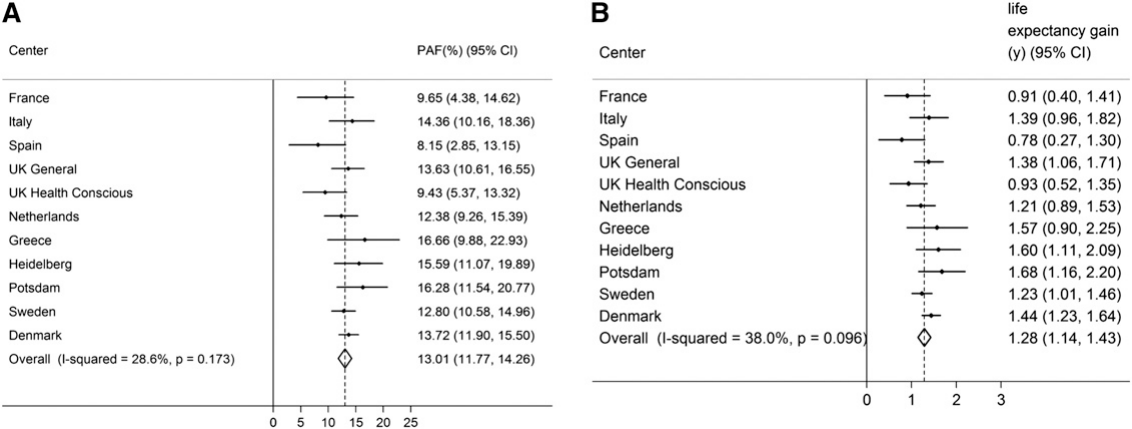
The combined proportion of number of deaths averted when all inactivity (lowest category of Cambridge Index) and abdominal obesity (≥88 cm and ≥102 cm in women and men, respectively) were avoided (A) and estimated life expectancy gain when all inactivity and abdominal obesity were avoided (B). Data were adjusted for age, sex, education, smoking, and alcohol intake (*n* = 334,161). PAF, population attributable fraction.

If all inactive individuals were at least moderately inactive, the number of deaths would theoretically be reduced by 7.35% (95% CI: 5.88, 8.83; *I*^2^: 70.2%; *P* < 0.001) (**Figure 1A**), and life expectancy at birth would increase by 0.70 y (95% CI: 0.56, 0.84). These estimates were only marginally attenuated by additional adjustment for BMI (*I*^2^: 70.7%; PAF: 7.08%; 95% CI: 5.58, 8.58). Comparable estimates for obesity (BMI >30) were lower: 3.66% (95% CI: 2.30, 5.01; *I*^2^: 82.5%; *P* < 0.001; Figure 1B) and 0.34 y (95% CI: 0.21, 0.48), which suggests that physical inactivity is responsible for more than twice as many deaths as general obesity in this European population. Finally, we calculated the PAF and gain in life expectancy for avoiding high WC (>88 cm and ≥102 cm in women and men, respectively), and the estimate was similar to that for inactivity: 6.53% (95% CI: 4.90, 8.15) (Figure 1C), corresponding to an estimated gain in life expectancy of 0.62 y (95% CI: 0.46, 0.79). The combined PAFs and estimated gains in life expectancy for inactivity and BMI and high WC are shown in **Figure 2**A, B and **Figure 3**A, B. **Supplemental Table 7** shows the proportion of deaths exposed to physical inactivity, general obesity, and abdominal obesity and the respective adjusted RRs by study center.

## DISCUSSION

PA is inversely associated with all-cause mortality at all levels of BMI and WC. The greatest reduction in risk was observed in the comparison of inactive and moderately active groups. Physical inactivity may theoretically be responsible for twice as many total deaths as high BMI (≥30) in this population, similar to the number of deaths averted if abdominal adiposity were eliminated.

We observed significant interactions between PA and BMI and WC in relation to all-cause mortality. The most pronounced risk reductions by increasing levels of PA were observed in those categorized as normal weight and abdominally lean. However, across all strata for both general and abdominal adiposity, a markedly reduced hazard was observed between those categorized as inactive and those categorized as moderately inactive. Data from the United States suggest that PA reduces but does not eliminate the increased risk of adiposity on all-cause mortality when cross-classifying activity and BMI groups (5, 6). Others have shown that exercising for 15 min/d (defined as the low-volume exercise group) is associated with a 14% reduction in risk of all-cause mortality compared with inactivity in an Asian population (2). Our results extend these previous observations to also include European men and women and suggest that, within each strata for BMI and WC, the hazard of all-cause mortality was substantially reduced when the inactive group was compared with the moderately inactive group. Thus, emerging evidence is accumulating indicating that substantial health benefits may be achieved by fairly small increases in PA.

Results from our calibration study suggest that moving from one category to the next (e.g., from the inactive to the moderately inactive group) is associated with an increase in PAEE of between 90 and 110 kcal/d in men and women with a similar BMI, as in the EPIC cohort (16). Assuming that all inactive individuals were truly inactive and did not participate in moderate and vigorous intensity, this amount of energy expenditure can be achieved by an increase in PAEE equivalent to ∼20 min of brisk walking per day, which is lower than the current PA recommendations for public health (22–24).

Approximately 9.2 million deaths occurred in European men and women in 2008 (25), of which—according to our estimates from the current study—676,000 deaths may be attributable to physical inactivity compared with 337,000 deaths attributable to obesity (BMI >30). Our PAF estimates were derived directly from the RR estimates in the EPIC population, prevalence data using measured exposures and based on theoretically avoiding high BMI (≥30), and all physical inactivity (the inactive category from the Cambridge Index)—more likely achievable in population-wide public health campaigns.

Our estimate of gain in life expectancy for PA is similar to that from Lee et al. (3) but lower than that from other comparable studies (2, 4, 26). The differences between studies are likely explained by differences in the assessment and the prevalence of the risk factors and RRs between populations, different calculations of PAFs, and differences in subsequent gains in life expectancy. In this cohort, the proportions of people at risk were 22.7% for inactivity, 15.8% for obesity, and 21.9% for high WC. These prevalence estimates, and hence the estimates of PAF, may differ between populations. However, the advantage of examining the PAFs for these risk factors within a single cohort using the observed prevalence estimates is the ability to compare the relative importance of physical inactivity and central and general obesity. Our results suggest that the influence of physical inactivity on mortality appears to be greater than that of high BMI and similar to that of high WC in European men and women.

Our results should be interpreted keeping the following limitations in mind. We observed significant heterogeneity in PAF estimates across centers. Apart from differences in RR estimates and the prevalence of risk factors between centers, heterogeneity was mainly explained by the Greek center (in men), where the risk of death associated with general and abdominal obesity was inverse in combination with the highest PAF for physical inactivity; by the French cohort (women only), in which neither general nor abdominal obesity was associated with an increased risk of premature death; and by the Spanish center (in women), in which no association between PA and mortality was observed.

Furthermore, whereas BMI and WC may both be indicators of total fat mass rather than general and abdominal adiposity, respectively (27), prospective observational studies consistently suggest a higher risk of mortality associated with high WC than with high BMI (28–30). From a public health perspective, it is therefore encouraging that our results suggest that small increases in PA in those who are currently categorized as inactive appear to be associated with significant reductions in all-cause mortality at all levels of BMI and WC.

The strengths of this study included its prospective design and its large number of participants, for whom measured height, weight, and WC values spanned a wide range. The large sample size and long follow-up facilitated exclusion of early years of follow-up in the sensitivity analysis. This minimized the likelihood of confounding resulting from low activity in lean participants with subclinical disease. A broad range of covariates permitted the evaluation of potential confounders, such as diet, smoking history, and alcohol intake. HRs, PAFs, and gains in life expectancy were estimated by using a validated measure of PA (16). Obesity was defined on the basis of measured height, weight, and WC, which eliminated misclassification bias. Although our global measure of PA has been validated (16), it is still a relatively imprecise way of characterizing activity. However, the impact of nondifferential misclassification would be to attenuate the association between activity and mortality. Thus, our results are more likely to underestimate the true importance of PA rather than to accentuate it. The same is not true for obesity, because it is more accurately measured; thus, the estimates of the association reported here are more likely to reflect the true underlying association.

EPIC Europe is a population-based study, but it was never intended to be entirely representative of the adult European population. Representativeness matters considerably in attempts to generalize prevalence estimates of an exposure or a disease, but it is much less of an issue in the interpretation of measures of association in a cohort study, provided the full range of exposures is observed in the population. Despite not being entirely representative, the prevalence of obesity in our sample is similar to that reported for European men and women (31). We considered the possibility of confounding by co-existent disease among participants by adjusting for comorbidities and excluding deaths in the first 3 y of follow-up. PA and anthropometric measures were assessed only at baseline; therefore, the current analysis did not take into consideration changes in PA and anthropometric measures over time.

The greatest reductions in all-cause mortality risk were observed between the inactive and the moderately inactive groups across levels of general and abdominal adiposity, which suggests that efforts to encourage even small increases in activity in inactive individuals may be of public health benefit. The hypothetical number of deaths reduced by avoiding inactivity in this population may be double that with an approach that avoided high BMI and similar to that of an approach that avoided high WC.

## Supplementary Material

Supplemental data

## Footnotes

7 Abbreviations used: EPIC, European Prospective Investigation into Cancer and Nutrition; PA, physical activity; PAEE, physical activity energy expenditure; PAF, population attributable fraction; WC, waist circumference.
