# Supplementary material for: Physical activity and all-cause mortality across levels of overall and abdominal adiposity in European men and women: the European Prospective Investigation into Cancer and Nutrition Study (EPIC)1
Source: Am J Clin Nutr. 2015 Jan 14;101(3):613–21. doi: 10.3945/ajcn.114.100065 (PMC4340064; doi:10.3945/ajcn.114.100065)
Supplement: Supplemental data [file supp_101_3_613__index.html]

Physical activity and all-cause mortality across levels of overall and abdominal adiposity in European men and women: the European Prospective Investigation into Cancer and Nutrition Study (EPIC) — Physical activity and all-cause mortality across levels of overall and abdominal adiposity in European men and women: the European Prospective Investigation into Cancer and Nutrition Study (EPIC) — Supplemental data 

# Physical activity and all-cause mortality across levels of overall and abdominal adiposity in European men and women: the European Prospective Investigation into Cancer and Nutrition Study (EPIC)

## Supplemental data

**Files in this Data Supplement:**

- Supplemental data - Tables 1-7
