# Supplementary material for: Physical activity and all-cause mortality across levels of overall and abdominal adiposity in European men and women: the European Prospective Investigation into Cancer and Nutrition Study (EPIC)1
Source: Am J Clin Nutr. 2015 Jan 14;101(3):613–21. doi: 10.3945/ajcn.114.100065 (PMC4340064; doi:10.3945/ajcn.114.100065)
Supplement: Supplemental data [file 114.100065_ajcn100065SupplementaryData1.docx]

**Physical Activity and all-cause mortality across levels of general and abdominal adiposity: The European Prospective Investigation into Cancer and Nutrition Study (EPIC)**

Ulf Ekelund*^1,2^, Heather A Ward*^3^, Teresa Norat^3^, Jian’an Luan^1^, Anne M May^4^, Elisabete Weiderpass^5-8^, Stephen S Sharp^1^, Kim Overvad^9,10^, Jane Nautrup Østergaard^9,10^ Anne Tjønneland^11^, Nina Føns Johnsen^11^, Sylvie Mesrine^12,13,14^, Agnès Fournier^12,13,14^, Guy Fagherazzi^12,13,14^, Antonia Trichopoulou^15,16^, Pagona Lagiou^15,17,18^, Dimitrios Trichopoulos^16,17,18^, Kuanrong Li^19^, Rudolf Kaaks^19^, Pietro Ferrari^20^, Idlir Licaj^20^, Mazda Jenab^20^, Manuela Bergmann^21^, Heiner Boeing^21^, Domenico Palli^22^, Sabina Sieri^23^, Salvatore Panico^24^, Rosario Tumino^25^, Paolo Vineis^3,26^, Petra H Peeters^4^, Evelyn Monnikhof^4^, H.Bas Bueno-de-Mesquita^3,27,28^, J Ramón Quirós^29^, Antonio Agudo^30^, María-José Sánchez^31,32^, José María Huerta^32,33^, Eva Ardanaz^32,34^, Larraitz Arriola^35^, Bo Hedblad^36^, Elisabet Wirfält^37^, Malin Sund^38^, Mattias Johansson^38^, Timothy J Key^39^, Ruth C Travis^39^, Kay-Tee Khaw^40^, Søren Brage^1^, Nicholas J Wareham**^1^, Elio Riboli**^3^

^1^MRC Epidemiology Unit, University of Cambridge, UK

^2^Department of Sport Medicine, Norwegian School of Sport Sciences, Oslo, Norway

^3^Imperial Collage, London, UK

^4^University Medical Centre Utrecht, Julius Centre for Health Sciences and Primary Care, Utrecht, The Netherlands.

^5^Department of Community Medicine, Faculty of Health Sciences, University of Tromsø, Tromsø, Norway

^6^Department of Research, Cancer Registry of Norway, Oslo, Norway

^7^Department of Medical Epidemiology and Biostatistics, Karolinska Institutet, Stockholm, Sweden

^8^Samfundet Folkhälsan, Helsinki, Finland

^9^Section for Epidemiology, Department of Public Health, Aarhus University, Aarhus, Denmark

^10^Department of Cardiology, Center for Cardiovascular Research, Aalborg University Hospital, Aalborg, Denmark

^11^Danish Cancer Society, Copenhagen, Denmark

^12^Inserm, Centre for research in Epidemiology and Population Health (CESP), U1018, Nutrition, Hormones and Women’s Health team, Villejuif, France

^13^Univ Paris Sud, UMRS 1018, Villejuif, France

^14^IGR, Villejuif, France

^15^WHO Collaborating Center for Food and Nutrition Policies, Department of Hygiene, Epidemiology and Medical Statistics, University of Athens Medical School, Athens, Greece

^16^Hellenic Health Foundation, Athens Greece

^17^Department of Epidemiology, Harvard School of Public Health, Boston, USA

^18^Bureau of Epidemiologic Research, Academy of Athens, Athens, Greece

^19^Division of Cancer Epidemiology, German Cancer Research Centre, Heidelberg, Germany

^20^ IARC

^21^Department of Epidemiology, Deutsches Institut für Ernährungsforschung, Potsdam-Rehbrücke, Germany

^22^Molecular and Nutrional Epidemiology Unit, ISPO, Cancer Prevention and Research Institute, Florence, Italy

^23^Epidemiology and Prevention Unit, Fondazione IRCCS Istituto Nazionale dei Tumori, Milano, Italy

^24^Dipartimento di Medicina Clinica e Chirurgia, Federico ii University, Naples, Italy

^25^U.O.S. Registro Tumori e U.O.C. Anatomia Patologica , Ospedale “Civile M.P.Arezzo” ASP 7, Ragusa, Italy

^26^HuGEF Foundation, Turin Italy

^27^National Institute for Public Health and the Environment (RIVM), Bilthoven, The Netherlands

^28^Department of Gastroenterology and Hepatology, University Medical Centre, Utrecht, The Netherlands

^29^Public Health Directorate, Asturias, Spain

^30^Unit of Nutrition, Environment and Cancer, Cancer Epidemiology Research Program, Catalan Institute of Oncology

^31^Andalusian School of Public Health, Granada, Spain

^32^CIBER de Epidemiología y Salud Pública (CIBERESP), Spain.

^33^Department of Epidemiology, Murcia Regional Health Council, Murcia, Spain

^34^Navarre Public Health Institute, Pamplona, Spain

^35^Public Health Division of Gipuzkoa, Instituto BIO-Donostia, Basque Government, CIBER Epidemiología y Salud Pública-CIBERESP, Spain

^36^Cardiovascular epidemiology, Dept of Clinical Sciences, Lund University, Malmö, Sweden

^37^Nutritional Epidemiology, Dept of Clinical Sciences, Lund University, Malmö, Sweden

^38^Dept of Public Health and Clinical Medicine, Umeå University, Umeå, Sweden

^39^Cancer Epidemiology Unit, Nuffield Department of Clinical Medicine, University of Oxford, Oxford, UK

^40^Clinical Gerontology Unit, University of Cambridge, Cambridge, UK

*joint first authors

**these authors contributed equally to the work

**Supplementary Table 1** shows a detailed description of those excluded and comparison between excluded and included participants.

**Supplementary Table 2** shows the anthropometric and lifestyle characteristics stratified by sex.

**Supplementary Tables 3, 4 and 5** shows the associations between physical activity and all-cause mortality stratified by general and abdominal adiposity and by sex (Table 3), age group (Table 4) and smoking status (Table 5).

**Supplementary Tables 6** shows the association between recreational and occupational physical activity and all-cause mortality stratified by general and abdominal adiposity.

**Supplementary Table 7** shows the proportion of deaths exposed to the risk factor $\text{p}_{\text{d}}$ and adjusted relative risk (RR) by study center.

| **Supplementary Table 1**. Comparison of anthropometric and lifestyle characteristics among included and excluded EPIC participants in the present analysis | | | |
| --- | --- | --- | --- |
|  | Excluded (n=184247 ) | | Included (n=334161 ) |
|  | N missing | Mean (SD) | Mean (SD) |
|  |  |  |  |
| Age at recruitment | 0 | 51.2 (0.02) | 51.7 (0.02) |
| Length of follow-up | 0 | 13.0 (0.01) | 12.4 (0.001) |
| Waist circumference (cm) | 125918 | 83.6 (0.06) | 85.5 (0.02) |
|  | N missing | % | % |
| Proportion of male participants * | 0 | 19.2 | 35.0 |
|  |  |  |  |
| Cambridge physical activity index | 46308 |  |  |
| Inactive |  | 24.4 | 22.7 |
| Moderately inactive |  | 35.6 | 33.8 |
| Moderately active |  | 26.2 | 23.2 |
| Active |  | 13.8 | 20.3 |
|  |  |  |  |
| Education | 20828 |  |  |
| Primary school or less |  | 23.5 | 35.1 |
| Technical/Professional school |  | 19.6 | 24.9 |
| Secondary school |  | 32.4 | 15.6 |
| Longer education |  | 24.5 | 24.5 |
|  |  |  |  |
| Alcohol (g/d) | 6627 |  |  |
| 0 |  | 13.9 | 13.5 |
| >0-6 |  | 50.0 | 33.5 |
| >6-12 |  | 16.2 | 17.4 |
| <12-24 |  | 11.1 | 17.5 |
| >24 - 60 |  | 7.5 | 15.2 |
| >60 |  | 1.3 | 3.0 |
|  |  |  |  |
| Smoking status | 12414 |  |  |
| Never |  | 52.6 | 48.2 |
| Former |  | 26.9 | 27.7 |
| Smoker |  | 20.5 | 24.1 |
|  |  |  |  |
| BMI group | 98517 |  |  |
| < 18.5 |  | 6.5 | 0 |
| 18.5-24.9 |  | 46.6 | 45.9 |
| 25- 29.9 |  | 34.0 | 38.3 |
| >30 |  | 12.9 | 15.8 |
|  |  |  |  |
| Mortality events |  | 8.4 | 6.4 |

* Measured height and weight data were missing from 71% (n= 21,672) of participants from France, which is an all-female cohort

| **Supplementary Table 2. Anthropometric and lifestyle characteristics by sex** | | |
| --- | --- | --- |
|  | Male (n=116,980) | Female (n=217,181) |
|  | Mean (SD) | Mean (SD) |
| BMI (kg/m^2^) | 26.6 (3.6) | 25.6 (4.5) |
| Waist circumference (cm) | 94.8 (10.1) | 80.6 (11.4) |
|  | % | % |
| Cambridge physical activity index | |  |
| Inactive | 18.2 | 25.2 |
| Moderately inactive | 31.2 | 35.1 |
| Moderately active | 24.8 | 22.4 |
| Active | 25.7 | 17.4 |
|  |  |  |
| Education |  |  |
| Primary school or less | 35.2 | 35.1 |
| Technical/Professional school | 24.7 | 25.0 |
| Secondary school | 11.7 | 17.6 |
| Longer education | 28.4 | 22.3 |
|  |  |  |
| Alcohol (g/d) | |  |
| 0 | 6.5 | 13.5 |
| >0-6 | 20.4 | 33.5 |
| >6-12 | 16.2 | 17.4 |
| <12-24 | 22.2 | 17.5 |
| >24 - 60 | 27.2 | 15.2 |
| >60 | 7.5 | 3.0 |
|  |  |  |
| Smoking status |  |  |
| Never | 31.5 | 57.2 |
| Former | 37.3 | 22.5 |
| Smoker | 31.3 | 20.3 |
| Leisure time activity (hours/week) |  |  |
| 0 | 33.0 | 34.7 |
| <3.5 | 35.5 | 37.2 |
| 3.5 -7 | 18.7 | 17.9 |
| >7 | 12.8 | 10.2 |
|  |  |  |
| Occupational activity |  |  |
| Sitting | 37.5 | 26.8 |
| Standing | 20.7 | 22.5 |
| Manual work | 14.8 | 7.2 |
| Heavy manual work | 4.5 | 1.1 |
| Non-worker | 21.1 | 40.4 |
| Missing | 1.4 | 2.0 |

| **Supplementary Table 3.** The hazard ratio (HR and 95% CI) of all-cause mortality in relation to physical activity within strata of BMI and WC groups by sex | | | | | | | | | |
| --- | --- | --- | --- | --- | --- | --- | --- | --- | --- |
|  |  | | Inactive | Moderately inactive | Moderately active | Active | | |  |
|  | Deaths (no.) | | HR (95% CI) | HR (95% CI) | HR (95% CI) | HR (95% CI) | | |  |
| **Men**  **BMI (kg/m^2^)** | |  |  |  |  |  | | |  |
| 18.5-24.9 | | 3662 | 1 (reference) | 0.76(0.70-0.83) | 0.75(0.68-0.83) | 0.66(0.59-0.73) | | |  |
| 25 - 29.9 | | 5191 | 1 (reference) | 0.83(0.77-0.89) | 0.79(0.73-0.86) | 0.75(0.69-0.82) | | |  |
| >30 | | 2233 | 1 (reference) | 0.88(0.79-0.98) | 0.83(0.73-0.94) | 0.89(0.78-1.01) | | |  |
|  | |  |  |  |  |  | | |  |
| **WC (cm)** | |  |  |  |  |  | | |  |
| <88(W)/<102(M) | | 7545 | 1 (reference) | 0.80(0.76-0.85) | 0.78(0.73-0.84) | 0.72(0.67-0.77) | | |  |
| >=88(W)/>=102(M) | | 3541 | 1 (reference) | 0.87(0.80-0.95) | 0.82(0.74-0.91) | 0.86(0.77-0.96) | | |  |
|  | |  |  |  |  |  | | |  |
| **Women** | |  |  |  |  |  | | |  |
| **BMI (kg/m^2^)** | |  |  |  |  |  | | |  |
| 18.5- 24.9 | | 4623 | 1 (reference) | 0.79(0.73-0.85) | 0.72(0.65-0.78) | 0.68(0.62-0.76) | | |  |
| 25 - 29.9 | | 3624 | 1 (reference) | 0.81(0.74-0.88) | 0.77(0.69-0.86) | 0.76(0.68-0.85) | | |  |
| >30 | | 2105 | 1 (reference) | 0.81(0.72-0.90) | 0.70(0.61-0.81) | 0.75(0.64-0.89) | | |  |
|  | |  |  |  |  |  | | |  |
| **WC (cm)** | |  |  |  |  |  | | |  |
| <88(W)/<102(M) | | 6817 | 1 (reference) | 0.81(0.76-0.86) | 0.75(0.69-0.81) | 0.74(0.68-0.80) | | |  |
| >=88(W)/>=102(M) | | 3535 | 1 (reference) | 0.79(0.73-0.86) | 0.74(0.66-0.82) | 0.71(0.63-0.81) | | |  |
| Models adjusted for education, smoking, and alcohol; stratified by age at recruitment and study centre | | | | | | |  |  | |

| **Supplementary Table 4**. The hazard ratio (HR and 95% CI) of all-cause mortality in relation to physical activity levels within strata of BMI and WC groups by age group | | | | | | | | |
| --- | --- | --- | --- | --- | --- | --- | --- | --- |
|  |  | Inactive | Moderately inactive | Moderately active | Active | | |  |
|  | Deaths (no.) | HR (95% CI) | HR (95% CI) | HR (95% CI) | HR (95% CI) | | |  |
| **Under 60 years** |  |  |  |  |  | | |  |
| **BMI (kg/m^2^)** |  |  |  |  |  | | |  |
| 18.5-24.9 | 4082 | 1 (reference) | 0.81(0.74-0.88) | 0.74(0.68-0.82) | 0.69(0.63-0.76) | | |  |
| 25 - 29.9 | 3906 | 1 (reference) | 0.86(0.78-0.94) | 0.85(0.77-0.93) | 0.80(0.73-0.89) | | |  |
| >30 | 1946 | 1 (reference) | 0.91(0.81-1.03) | 0.80(0.70-0.92) | 0.87(0.76-0.99) | | |  |
|  |  |  |  |  |  | | |  |
| **WC (cm)** |  |  |  |  |  | | |  |
| <88(W)/<102(M) | 7001 | 1 (reference) | 0.83(0.77-0.88) | 0.81(0.75-0.87) | 0.76(0.70-0.81) | | |  |
| >=88(W)/>=102(M) | 2933 | 1 (reference) | 0.91(0.83-1.00) | 0.79(0.71-0.88) | 0.84(0.75-0.94) | | |  |
|  |  |  |  |  |  | | |  |
| **60 years and older** |  |  |  |  |  | | |  |
| **BMI (kg/m^2^)** |  |  |  |  |  | | |  |
| 18.5- 24.9 | 4203 | 1 (reference) | 0.76(0.70-0.82) | 0.74(0.67-0.81) | 0.66(0.59-0.74) | | |  |
| 25 - 29.9 | 4909 | 1 (reference) | 0.81(0.75-0.87) | 0.75(0.69-0.82) | 0.73(0.66-0.81) | | |  |
| >30 | 2392 | 1 (reference) | 0.80(0.72-0.88) | 0.75(0.66-0.86) | 0.80(0.69-0.94) | | |  |
|  |  |  |  |  |  | | |  |
| **WC (cm)** |  |  |  |  |  | | |  |
| <88(W)/<102(M) | 7361 | 1 (reference) | 0.80(0.76-0.85) | 0.75(0.70-0.80) | 0.72(0.66-0.78) | | |  |
| >=88(W)/>=102(M) | 4143 | 1 (reference) | 0.79(0.73-0.85) | 0.80(0.72-0.89) | 0.77(0.68-0.86) | | |  |
| Models adjusted for sex, education, smoking, and alcohol; stratified by age at recruitment and study centre | | | | | |  |  | |

| **Supplementary Table 5**. The hazard ratio (HR and 95% CI) of all-Cause mortality in relation to physical activity levels within strata of BMI and WC groups by smoking status | | | | | | | |
| --- | --- | --- | --- | --- | --- | --- | --- |
|  |  | Inactive | Moderately inactive | Moderately active | Active | |  |
|  | Deaths (no.) | HR (95% CI) | HR (95% CI) | HR (95% CI) | HR (95% CI) | |  |
| **Never smoker** |  |  |  |  |  | |  |
| **BMI (kg/m^2^)** |  |  |  |  |  | |  |
| 18.5-24.9 | 2679 | 1 (reference) | 0.81(0.73-0.89) | 0.79(0.70-0.89) | 0.73(0.64-0.84) | |  |
| 25 - 29.9 | 2845 | 1 (reference) | 0.88(0.80-0.97) | 0.84(0.75-0.95) | 0.85(0.74-0.97) | |  |
| >30 | 1744 | 1 (reference) | 0.78(0.69-0.88) | 0.72(0.61-0.84) | 0.72(0.60-0.87) | |  |
|  |  |  |  |  |  | |  |
| **WC (cm)** |  |  |  |  |  | |  |
| <88(W)/<102(M) | 4662 | 1 (reference) | 0.85(0.78-0.91) | 0.81(0.74-0.88) | 0.79(0.71-0.87) | |  |
| >=88(W)/>=102(M) | 2606 | 1 (reference) | 0.82(0.74-0.91) | 0.80(0.70-0.91) | 0.81(0.70-0.94) | |  |
|  |  |  |  |  |  | |  |
| **Former smoker** |  |  |  |  |  | |  |
| **BMI (kg/m^2^)** |  |  |  |  |  | |  |
| 18.5- 24.9 | 2232 | 1 (reference) | 0.75(0.67-0.83) | 0.63(0.55-0.72) | 0.63(0.55-0.73) | |  |
| 25 - 29.9 | 3049 | 1 (reference) | 0.81(0.74-0.90) | 0.80(0.71-0.89) | 0.75(0.67-0.84) | |  |
| >30 | 1378 | 1 (reference) | 0.91(0.79-1.04) | 0.82(0.69-0.97) | 0.90(0.76-1.07) | |  |
|  |  |  |  |  |  | |  |
| **WC (cm)** |  |  |  |  |  | |  |
| <88(W)/<102(M) | 4372 | 1 (reference) | 0.81(0.75-0.88) | 0.75(0.68-0.82) | 0.77(0.69-0.85) | |  |
| >=88(W)/>=102(M) | 2287 | 1 (reference) | 0.85(0.77-0.95) | 0.78(0.69-0.89) | 0.76(0.66-0.87) | |  |
|  |  |  |  |  |  | |  |
| **Current smoker** |  |  |  |  |  | |  |
| **BMI (kg/m^2^)** |  |  |  |  |  | |  |
| 18.5- 24.9 | 3374 | 1 (reference) | 0.78(0.71-0.86) | 0.78(0.70-0.86) | 0.67(0.60-0.75) | |  |
| 25 - 29.9 | 2921 | 1 (reference) | 0.77(0.70-0.85) | 0.75(0.67-0.84) | 0.71(0.64-0.80) | |  |
| >30 | 1216 | 1 (reference) | 0.89(0.77-1.03) | 0.82(0.69-0.97) | 0.87(0.73-1.04) | |  |
|  |  |  |  |  |  | |  |
| **WC (cm)** |  |  |  |  |  | |  |
| <88(W)/<102(M) | 5328 | 1 (reference) | 0.78(0.73-0.84) | 0.78(0.72-0.84) | 0.68(0.63-0.75) | |  |
| >=88(W)/>=102(M) | 2183 | 1 (reference) | 0.85(0.76-0.95) | 0.79(0.70-0.90) | 0.84(0.73-0.96) | |  |
| Models adjusted for sex, education and alcohol; stratified by age at recruitment and study centre | | | | | |  | |

| **Supplementary Table 6**. The hazard ratio (HR and 95% CI) of all-cause mortality in relation to occupational and leisure time physical activity within strata of BMI and WC groups | | | | | | | | | | | | | | |
| --- | --- | --- | --- | --- | --- | --- | --- | --- | --- | --- | --- | --- | --- | --- |
|  |  | Occupational physical activity ^a^ | | | | | | | | | | | | |
|  | Deaths | Sitting  (n=102,133) | | Standing  (n=73,004) | | | Manual work  (n=32,924) | | | Heavy manual work  (n=7,679) | | | Non-worker  (n=112,499) | |
|  | (no.) | HR (95% CI) | | HR (95% CI) | | | HR (95% CI) | | | HR (95% CI) | | | HR (95% CI) | |
| **BMI (kg/m^2^)** |  |  |  | |  |  | | |  | |  |  | |  |
| 18.5-24.9 | 8,143 | 1 (reference) | | 1.01 (0.94 – 1.08) | | | 0.94 (0.86 – 1.03) | | | 0.97 (0.82 – 1.14) | | | 1.26 (1.17 – 1.35) | |
| 25 – 29.9 | 8,693 | 1 (reference) | | 1.07 (0.99 – 1.15) | | | 0.97 (0.89 – 1.07) | | | 0.96 (0.83 – 1.11) | | | 1.28 (1.20 – 1.37) | |
| >30 | 4,266 | 1 (reference) | | 1.13 (1.00 – 1.26) | | | 1.03 (0.90 – 1.17) | | | 1.11 (0.92 – 1.34) | | | 1.32 (1.19 – 1.46) | |
|  |  |  | |  | | |  | | |  | | |  | |
| **WC (cm)** |  |  | |  | | |  | | |  | | |  | |
| <88(W)/<102(M) | 14,132 | 1 (reference) | | 1.05 (0.99 – 1.11) | | | 0.96 (0.90 – 1.03) | | | 0.98 (0.87 – 1.10) | | | 1.28 (1.21 – 1.35) | |
| >88(W)/>102(M) | 6,970 | 1 (reference) | | 1.08 (0.99 – 1.18) | | | 1.00 (0.89 – 1.11) | | | 1.06 (0.91 – 1.24) | | | 1.33 (1.22 – 1.44) | |
|  |  |  | | | | | | | | | | | | |
|  |  | Recreational physical activity | | | | | |  | | | | | | |
|  | Deaths | 0 hours per week  (n=113,938) | | <3.5 hours per week (n=122,352) | | | 3.5-7 hours per week (n=60,773) | | | >7 hours per week  (n=37,098) | | |  | |
| **BMI (kg/m^2^)** | (no.) | HR (95% CI) | | HR (95% CI) | | | HR (95% CI) | | | HR (95% CI) | | |  | |
| 18.5-24.9 | 8,285 | 1 (reference) | | 0.76 (0.72 – 0.80) | | | 0.69 (0.64 – 0.73) | | | 0.73 (0.68 – 0.79) | | |  | |
| 25 – 29.9 | 8,815 | 1 (reference) | | 0.82 (0.78 – 0.87) | | | 0.79 (0.74 – 0.84) | | | 0.80 (0.74 – 0.87) | | |  | |
| >30 | 4,338 | 1 (reference) | | 0.84 (0.77 – 0.90) | | | 0.82 (0.74 – 0.91) | | | 0.80 (0.71 – 0.91) | | |  | |
|  | |  | |  | | |  | | |  | | |  | |
| **WC (cm)** |  |  | |  | | |  | | |  | | |  | |
| <88(W)/<102(M) | 14,362 | 1 (reference) | | 0.80 (0.77 – 0.84) | | | 0.75 (0.71 – 0.79) | | | 0.78 (0.73 – 0.83) | | |  | |
| >88(W)/>102(M) | 7,076 | 1 (reference) | | 0.82 (0.78 – 0.87) | | | 0.81 (0.75 – 0.88) | | | 0.81 (0.74 – 0.90) | | |  | |

^a^Occupational physical activity, 4 categories according to the ‘Cambridge index’

| Models adjusted for education, smoking, and alcohol; stratified by age at recruitment and study centre |  |  |
| --- | --- | --- |

| **Supplementary Table 7.** Proportion of deaths (standard error) exposed to the risk factor $\text{p}_{\text{d}}$ and adjusted relative risk (RR; [standard error]) by study center. | | | | | | | | | | |
| --- | --- | --- | --- | --- | --- | --- | --- | --- | --- | --- |
| EPIC center | | | | | **Inactivity** | | **General Obesity** | | **Abdominal Obesity** | |
|  | |  | | | $\text{p}_{\text{d}}$ | RR | $\text{p}_{\text{d}}$ | RR | $\text{p}_{\text{d}}$ | RR |
| France | |  | | | 0.15 (0.01) | 1.20 (0.14) | 0.28 (0.02) | 1.4 (0.13) | 0.06 (0.01) | 0.95 (0.17) |
| Italy | |  | | | 0.29 (0.01) | 1.51 (0.09) | 0.36 (0.01) | 1.25 (0.07) | 0.20 (0.01) | 1.35 (0.09) |
| Spain | |  | | | 0.49 (0.01) | 1.16 (0.06) | 0.36 (0.01) | 1.07 (0.05) | 0.38 (0.01) | 1.19 (0.06) |
| UK | General | | | | 0.26 (0.01) | 1.26 (0.03) | 0.55 (0.01) | 1.22 (0.04) | 0.15 (0.01) | 1.16 (0.04) |
|  | Health Conscious | | | | 0.12 (0.01) | 1.42 (0.08) | 0.36 (0.02) | 1.24 (0.07) | 0.08 (0.01) | 1.19 (0.11) |
| Netherlands | | | |  | 0.32 (0.01) | 1.38 (0.07) | 0.15 (0.01) | 1.48 (0.10) | 0.16 (0.01) | 1.26 (0.08) |
| Greece | | |  | | 0.48 (0.01) | 1.01 (0.04) | 0.70 (0.01) | 1.31 (0.07) | 0.36 (0.01) | 1.05 (0.05) |
| Germany | | | Heidelberg | | 0.36 (0.02) | 1.42 (0.09) | 0.19 (0.01) | 1.52 (0.11) | 0.26 (0.01) | 1.46 (0.10) |
|  | | | Potsdam | | 0.33 (0.01) | 1.32 (0.08) | 0.34 (0.01) | 1.41 (0.09) | 0.25 (0.01) | 1.33 (0.09) |
| Sweden | | |  | | 0.23 (0.01) | 1.39 (0.04) | 0.33 (0.01) | 1.32 (0.04) | 0.15 (0.01) | 1.26 (0.05) |
| Denmark | | |  | | 0.31 (0.01) | 1.42 (0.04) | 0.18 (0.01) | 1.52 (0.05) | 0.19 (0.01) | 1.43 (0.05) |
| **Overall** | | |  | | 0.29 (0.003) | 1.31 (0.05) | 0.32 (0.003) | 1.33 (0.04) | 0.18 (0.003) | 1.25 (0.04) |
|  | | | **I^2^ (%)** | | 99.0 | 85.9 | 99.7 | 81.2 | 99.0 | 78.9 |
